# Therapeutic potential of HDAC6 inhibitor Tubastatin A in health and diseases: current perspective and future directions

**DOI:** 10.1016/j.mmr.2026.100024

**Published:** 2026-04-17

**Authors:** Sonu Rajput, Sumeet Kumar Singh, Poonam Yadav, Amit Khurana, Ralf Weiskirchen, Umashanker Navik

**Affiliations:** a Department of Pharmacology, Central University of Punjab, Bathinda 151401, Punjab, India; b Institute of Molecular Pathobiochemistry, Experimental Gene Therapy and Clinical Chemistry (IFMPEGKC), RWTH Aachen University Hospital, D-52074 Aachen, Germany

**Keywords:** Tubastatin A (Tub A), Histone deacetylase 6 (HDAC6), Age-related diseases, Neurodegenerative diseases, Diabetes, Cancer

## Abstract

Histone deacetylase 6 (HDAC6) is a unique, predominantly cytoplasmic enzyme that regulates a broad spectrum of cellular and physiological processes, including cell proliferation, migration, intracellular transport, and differentiation. Its distinct structural configuration, comprising two catalytic deacetylase domains and a zinc finger ubiquitin-binding domain (ZnF-BUZ), enables HDAC6 to deacetylate a variety of non-histone substrates, such as α-tubulin, heat shock protein 90 (Hsp90), cortactin, and peroxiredoxin (Prdx). Furthermore, HDAC6 plays a key role in cellular stress responses and cell survival by facilitating the clearance of misfolded proteins, inducing autophagy, and modulating the unfolded protein response. Despite its cytoprotective roles, HDAC6 has emerged as a therapeutic target due to its involvement in multiple pathological pathways and age-related disorders. Tubastatin A (Tub A), a novel and highly selective HDAC6 inhibitor, demonstrates strong therapeutic potential against neurodegenerative, cardiovascular, autoimmune, metabolic, cancer, and other diseases. Tub A enhances the acetylation of both histone and non-histone proteins, thereby modulating gene expression and diverse cellular processes. It shows pharmacological effects, including anti-inflammatory, neuroprotective, anti-diabetic, anti-obesity, anti-oxidant, and other activities. Moreover, preclinical evidence suggests that Tub A effectively regulates multiple pathological pathways by inhibiting HDAC6, which contributes to ameliorating age-related disorders. Therefore, Tub A represents a promising epigenetic modulator with broad therapeutic relevance. Hence, further comprehensive and large-scale investigations are warranted to elucidate its clinical potential and its roles in disease management, as no clinical data related to Tub A activity are available. This review highlights the therapeutic potential of the selective HDAC6 inhibitor Tub A across various pathological conditions, discusses current preclinical findings, and outlines key challenges and future directions for clinical translation.

## 1 Background

Epigenetics refers to heritable structural and biochemical changes in chromatin that result in molecular modifications of DNA that regulate gene activity without affecting the DNA sequence. Epigenetic modifications are induced by several factors, such as dietary intake, health conditions, or lifestyle modifications, in many physiological and pathological processes [1], [2], [3]. Meanwhile, aberrant epigenetic alterations affect gene expression by turning genes on or off, which may lead to the development of diseases, such as neurodegenerative, cancer, cardiovascular, autoimmune, metabolic, and other diseases [3], [4], [5], [6]. Epigenetic modifications involve various mechanisms, including DNA methylation, microRNAs (miRNAs) mediated regulation, and histone alterations that also include modifications at the post-translational level, such as acetylation, phosphorylation, and ubiquitination, among others. Histone deacetylation, mediated by histone deacetylases (HDACs), is one of the key modifications that removes acetyl groups from the ε-amino groups of lysine residues within histone and non-histone proteins [6], [7], [8]. Simultaneously, the post-translational mechanisms associated with histone modifications lead to changes in the N-terminal tails of histones [2], [7]. This enzymatic activity results in the condensation of chromatin structure, subsequently leading to the repression of gene expression [9], [10]. Moreover, deacetylation also plays a significant role in influencing cell differentiation, proliferation, and various physiological processes [11].

Chromatin consists of negatively charged DNA wrapped around nucleosomes, which contain the eight positively charged histones, two sets each of H2A, H2B, H3, and H4 [10], [12]. HDACs are categorized into 4 distinct classes: Class I (HDAC1, HDAC2, HDAC3, and HDAC8), Class II (HDAC4, HDAC5, HDAC6, HDAC7, HDAC9, and HDAC10), Class III (SIRT1, SIRT2, SIRT3, SIRT4, SIRT5, SIRT6, and SIRT7), and Class IV (HDAC11), all these enzymes play significant roles in various pathological conditions [13]. Except for Class III HDAC, all other HDAC classes possess zinc-binding capabilities, enabling them to hydrolyze the acetyl group. Moreover, Class II HDAC enzymes are further subdivided into subclasses IIa and IIb. Members of subclass IIb include HDAC6 and HDAC10, which are involved in the regulation of cytoskeletal functions and protein degradation in both the cytoplasm and the nucleus [14].

This review primarily focuses on HDAC6, which plays a key role in cellular stress responses and cell survival by facilitating the clearance of misfolded proteins, inducing autophagy, and modulating the unfolded protein response. It is a unique member of the Class IIb HDAC family, due to its distinct structural features and diverse biological functions. HDAC6 contains two homologous, functionally independent catalytic domains, CD1 and CD2, that contribute to its enzymatic activity. HDAC6 has a zinc finger ubiquitin-binding domain (ZnF-BUZ), which enables it to deacetylate a variety of non-histone substrates as well as misfolded-protein degradation through ubiquitination [15]. Unlike other HDACs, which are mainly located in the nucleus, HDAC6 is primarily found in the cytoplasm [16], [17], [18]. In addition to deacetylation of histone, HDAC6 also targets several non-histone proteins, such as heat shock protein 90 (Hsp90), α-tubulin, cortactin, heat shock factor 1, E2F, p53, and myogenic differentiation [16]. Interestingly, HDAC6 has 1216 amino acid residues and is also the largest member of the HDAC family [19]. Furthermore, HDAC6 expression is upregulated in diabetic nephropathy. Pharmacological inhibition of zinc-dependent catalytic activity of HDAC6 using ACY1215 or genetic silencing through siRNA effectively suppresses the transforming growth factor-β (TGF-β)/Smad and nuclear factor κB (NF-κB) signaling pathways, leading to a reduction in renal fibrosis and inflammation [20]. HDAC6 promotes the activities of proinflammatory mediators in macrophages through reactive oxygen species (ROS)-dependent mitogen-activated protein kinase (MAPK)-NF-κB/activator protein-1 (AP-1) signaling pathway [21]. Moreover, HDAC6 increases the levels of tumor necrosis factor-α (TNF-α), interleukin (IL)-1β, and IL-6, along with decreased levels of acetylated α-tubulin, highlighting HDAC6 as a potential molecular target for inflammatory regulation [21]. Additionally, HDAC6 deacetylates forkhead box O1 (FOXO1) and inhibits its transcriptional activity, thus destabilizing the expression of IL-17-producing helper T cells. Moreover, it is also linked with the activation of nucleotide-binding oligomerization domain, leucine-rich repeat, and pyrin domain containing 3 (NLRP3) inflammasome by regulating upstream and downstream signaling factors, such as activation of pro-IL-1β, p65 subunit, and MyD88, leading to further inflammation and ROS generation [22], [23].

Interestingly, Tubastatin A (Tub A) is a newly synthesized potent and highly selective HDAC6 inhibitor with over 1000-fold selectivity against all other isoforms except HDAC8 (57-fold). It is a promising therapeutic agent that targets HDAC6 and belongs to novel Class IIb HDAC6 inhibitors [24], [25]. Studies have shown that Tub A has therapeutic potential in preclinical studies for various conditions such as neurodegenerative diseases, cancer, cardiovascular issues, autoimmune diseases, and metabolic diseases, among others [16], [26], [27]. It targets multiple biological pathways, emphasizing its versatility in various therapeutic applications [25]. Therefore, in this review, we discuss the therapeutic potential of Tub A for age-related disorders, detailing its mechanisms and future implications.

## 2 Pharmacokinetics and pharmacodynamics of Tubastatin A

Pharmacokinetic and pharmacodynamic studies provide essential insights into the pharmacological characteristics of Tub A as a potential therapeutic agent. A study by Shen *et al*. [25] reported that the plasma half-life of Tub A after intravenous and oral administration in mice was less than 1 h (t_1/2_ = 30 min). In contrast, the half-life of Tub A in the microsomes and hepatocytes was relatively high, indicating better stability than that of the plasma [25]. Additionally, the high efflux ratio of Tub A suggests its low gastrointestinal (GI) absorption and oral bioavailability. Thus, the intraperitoneal (i.p.) route is the preferred administration choice in rodents [25], [28], [29]. Shen *et al*. [25] reported that the pharmacokinetics for the oral (30 mg/kg) dose of Tub A has area under curve (AUC, 134 h × ng/ml) and t_1/2_ (0.86 h), while through intravenous administration (3 mg/kg) reported with AUC (227 h × ng/ml) and t_1/2_ (0.35 h) in the plasma of CD1 mice. Furthermore, Tub A can cross the blood-brain barrier (BBB); the brain-to-plasma (B/P) ratio after intravenous administration (3 mg/kg) was 0.15 and 0.86 at 8 min and 60 min, respectively. Tub A has an uptake rate of <100 ng/g, with a clearance of 222 ml/(min·kg), and a steady-state volume of distribution of 4.14 L/kg [25]. According to Butler *et al*. [30], Tub A exhibits enhanced potency and selectivity due to the presence of a rigid tetrahydro-γ-carboline capping group covalently linked to a hydroxamic acid via a hydrophobic benzyl linker, resulting in significant inhibitory activity and high selectivity against HDAC6 [30]. Tub A is well tolerated when used either alone or in combination with other medications. Synthesized through structure-based drug design and homology modelling, Tub A, a tetrahydro-γ-carboline analogue, shows specificity for HDAC6 with a half maximal inhibitory concentration (IC_50_) of 15 nmol/L, more than 1000-fold selectivity compared to HDAC1, protecting against oxidative stress and potentially beneficial for treating autoimmune conditions [19]. A modified version of Tub A, with an enhanced rigid tetrahydro-β-carboline capping group, shows potent inhibitory actions targeting HDAC6 with an IC_50_ of 3.73 nmol/L [31].

Tub A demonstrates favourable bioavailability *in vivo*, meeting a key requirement for clinical trial development. However, further clinical research is necessary to evaluate this compound, as other HDAC inhibitors have shown adverse effects when targeting chronic conditions [32]. Comparing with clinically advanced HDAC6 inhibitors, such as ACY-1215 (IC_50_ of 4.7 nmol/L) [33], [34], ACY-241 (IC_50_ of 2.6 nmol/L) [35], and KA2507 (IC_50_ of 2.5 nmol/L) [36], the IC_50_ of Tub A (3.73 nmol/L) is slightly lower than that of ACY-1215; its plasma half-life (0.35 h) in mice is shorter than ACY-1215 (1.17 h) [33], [34] and KA2507 (1.6–4.9 h) [36], despite relatively high microsomal or hepatocyte stability. Nevertheless, based on pharmacokinetic and pharmacodynamic studies, Tub A shows potential to target multiple diseases by modulating different pathways, suggesting beneficial therapeutic effects in future clinical studies.

## 3 Tubastatin A in diseases

### 3.1 Neurodegenerative diseases

The gradual loss of structure and function of neurons in the central nervous system leads to a diverse group of disorders, such as neurodegenerative diseases. These include Alzheimer’s disease (AD), Parkinson’s disease (PD), Huntington’s disease, amyotrophic lateral sclerosis, and other related disorders [37]. Several studies have shown that various risk factors can contribute to neurodegenerative diseases by disrupting the autophagy process and mitochondrial function [38], [39]. This disruption can result in inflammation, oxidative stress, genetic and epigenetic abnormalities, reduced growth factor levels, impaired signaling pathways, and ultimately neuronal cell death [40]. Notably, HDAC6 has emerged as a potential therapeutic target due to its involvement in many of these pathogenic processes, thus placing it as a key regulator in the central nervous system and brain-related diseases [41], [42]. Processes such as cell proliferation, migration, intracellular transport, and cell differentiation are controlled by HDAC6 [43]. This section provides an overview of HDAC6’s role in different types of neurodegenerative diseases and discusses the therapeutic role of the selective HDAC6 inhibitor Tub A.

#### 3.1.1 Parkinson’s disease

PD is a neurodegenerative disorder characterized by the accumulation of α-synuclein, as well as aggregated and misfolded proteins. In PD models, the role of HDAC6 has been identified, with evidence showing that HDAC6 deacetylates non-histone substrates such as α-tubulin, Hsp90, and peroxiredoxin (Prdx) 1 and 2 [44], [45]. Additionally, a study by Jian *et al*. [46] reported a significant increase in HDAC6 expression in dopaminergic neurons in the 6-hydroxydopamine (6-OHDA) administration. The acetylation levels of Prdx1 and Prdx2 decreased due to the overexpression of HDAC6. Tub A inhibits HDAC6 expression in mice when administered at a dose of 25 mg/kg via the i.p*.* route for 7 d after the 6-OHDA-induced PD model. Tub A treatment enhanced the acetylation of Prdx1 and Prdx2, resulting in reduced production of ROS and inflammatory cytokines, leading to beneficial effects on anti-oxidant and anti-inflammatory activities, thus mitigating dopaminergic neurotoxicity in PD [46]. An *in vitro* experiment conducted by Yan *et al*. [47] using the SH-SY5Y human neuroblastoma cell line, neurotoxicity was developed using 6-OHDA. Its administration leads to increased expression of NLRP3 inflammasome and cell apoptosis. The treatment with Tub A at a dose of 15 µmol/L inhibited the deacetylation activity of HDAC6, provided an anti-apoptotic effect, reversed the NLRP3 inflammatory response, and protected dopaminergic neurons against 6-OHDA-induced damage [47]. The results of targeting HDAC6 activity through Tub A for the treatment of PD, shown in this study, still require further investigation utilizing a series of alternative approaches.

Furthermore, Francelle *et al*. [44] and Du *et al*. [48] reported that increased HDAC6 expression leads to PD by promoting the aggregation of α-synuclein and other proteins, partly by regulating α-synuclein oligomer levels through chaperone-mediated autophagy (CMA) activation. In adult female Wistar rats, Francelle *et al*. [44] found that Tub A (15 mg/kg) via the i.p. route for two weeks effectively suppressed the expression of a harmful variant of α-synuclein characterized by astrocyte reactivity and phosphorylation at the serine 129 position. Treatment with Tub A also protected dopaminergic neurons in the substantia nigra of the rats, leading to the upregulation of key members of CMA, thereby increasing protein acetylation, reducing neuroinflammation, and rescuing PD-related pathological pathways. Similarly, Du *et al*. [48] found that the treatment with Tub A (25 μg/g) via i.p. injection for 20 consecutive days inhibited HDAC6 levels and increased acetylated α-tubulin levels in a lactacystin-induced PD mouse model. Together, these studies indicate that inhibiting HDAC6 levels with Tub A significantly improves PD-related symptoms in rodent models.

Additionally, Pinho *et al*. [49] exposed zebrafish larvae to 1-methyl-4-phenylpyridinium (MPP+), a neurotoxic compound that selectively targets dopaminergic neurons, particularly in the substantia nigra region of the brain. This elevated the expression of HDAC1 and HDAC6, leading to altered behavioural and metabolic phenotypes in a zebrafish model of PD. However, the HDAC6 expression was inhibited after treatment with Tub A at a concentration of 1 µmol/L for 3−5 d post-fertilization. This study found that MS-275 inhibits HDAC1-mediated deacetylation of histone H3K9, whereas Tub A selectively blocks HDAC6-dependent tubulin K40 deacetylation, leading to enhanced expression of anti-oxidative genes and protection against ROS [49]. This study revealed that the HDAC6 inhibitor (Tub A) plays a vital role in attenuating PD within the zebrafish model.

Exogenous X-box binding protein 1 (XBP1), a major regulator of the unfolded protein response, has been reported to have protective effects against PD by enhancing neuronal survival and reducing oxidative stress. According to Zhang *et al*. [50], increased HDAC6 expression reduces XBP1 levels, leading to increased oxidative stress. The HEK293T cells were co-transfected with Flag-XBP1s and HA-Ub plasmids, then treated with Tub A at a concentration of 3 µmol/L. Results showed that the Tub A treatment reduces HDAC6 and increases XBP1 expression. These ultimately enhance the expression of anti-oxidative genes through the acetylation-mediated proteasomal degradation pathway [50].

#### 3.1.2 Giant axonal neuropathy and glioma

Further studies have also shown that increased HDAC6 expression triggers Toll-like receptor 4 (TLR4) activation, leading to decreased Sigma-1 receptor (Sig1R) expression via the TLR4/transforming growth factor-beta-activated kinase 1 (TAK1)/p38 MAPK signaling pathway, which is involved in microglial dysfunction associated with neuroinflammation [51], [52]. Pretreatment with Tub A at 1 μmol/L prevented the downregulation of *Sig1R* gene expression induced by lipopolysaccharide (LPS), inhibiting HDAC6 and blocking the TLR4/TAK1/p38 MAPK signaling pathway. This suggests that Tub A could be a potential therapy for neuroinflammatory diseases [51]. A separate study by Nath *et al*. [53] demonstrated that treating dorsal root ganglia (DRG) neurons and HEK293 cells with Tub A resulted in an increased level of acetylated α-tubulin, restoring lysosomal transport and rescuing axonal transport of organelles in cultured neurons. In the same study, on Gan^−/−^;TgPer mice, daily i.p. administration of Tub A at a dosage of 25 mg/kg for 4 weeks significantly inhibited HDAC6 overexpression, leading to increased tubulin acetylation levels and reducing the abnormal accumulation of peripherin and neurofilament proteins in spinal neurons. Tub A is therefore considered a potential treatment for giant axonal neuropathy (GAN) [53]. Furthermore, Li *et al*. [54] found that Tub A regulates the p97/VCP-facilitated ubiquitin-proteasome system (UPS) degradation pathway by inhibiting HDAC6 overexpression. This inhibition disrupts the aggresome-autophagy pathway, reducing the clearance of aggregated, ubiquitinated proteins. This study shows that targeting HDAC6 expression could be a promising strategy for treating glioma.

#### 3.1.3 Alzheimer’s disease

AD is a progressive neurological disorder that leads to dementia through memory loss and cognitive decline. The accumulation of amyloid β (Aβ) plaques, neurofibrillary tangles, and the deposition of tau proteins caused by hyperphosphorylation further leads to the loss of neuronal cells and synapses, which are the pathological hallmarks of AD [55]. Investigations have reported noticeable increases in HDAC6 levels in the hippocampus and cortex of humans and animals, which are implicated in AD development [56]. These two brain regions are crucial for recalling and learning information [57]. Most research studies suggest that elevated HDAC6 expression may facilitate AD-associated neurodegeneration, and modulation by HDAC6 inhibitors could be a potential treatment for AD [58], [59]. For example, a study conducted by Zhang *et al*. [60] found that increased HDAC6 levels lead to protein misfolding and the formation of aggregates such as Aβ and p-tau by regulating autophagy via the Akt/glycogen synthase kinase 3β (GSK3β)/mammalian target of rapamycin (mTOR) signaling pathway. The study’s findings indicated that Tub A (50 mg/kg; i.p. for 20 d) potentially inhibits the expression of HDAC6, resulting in mitigation of tau pathology by reducing tau phosphorylation and enhancing autophagy-related degradation through the Akt/GSK3β/mTOR signaling pathway, thereby ameliorating the cognitive deficits in a mouse model of AD [60].

Moreover, Selenica *et al*. [57] reported that Tub A (25 mg/kg; i.p. for 2 months in mice) administration significantly inhibits the overexpression of HDAC6 and enhances the acetylation level of α-tubulin. Further increased acetylation of Hsp90 ultimately reduces total tau levels and, consequently, improves behavioural deficits by targeting tau pathology. The findings of this study demonstrate that, in future investigations, the modulation of total tau levels through α-tubulin and other mechanisms will significantly influence behavioral phenotypes. The overall study indicates that inhibiting HDAC6 expression through Tub A may present potentially beneficial therapeutic strategies for AD and other neurodegenerative conditions.

*In vitro* studies showed that Tub A, at different concentrations ranging from 0.1 nmol/L to 1 μmol/L, mitigated the reduction in exogenous fibroblast growth factor 21 (FGF-21) expression, providing neuroprotection and restoring mitochondrial trafficking in rat cortical neurons following glutamate-induced excitotoxicity [29]. Another study demonstrated that the co-aggregation of apolipoprotein E4 (APOE4) and Aβ_1-40_ significantly reduced the level of choline acetyltransferase (ChAT) and altered the phosphorylated form of tau and GSK3β protein in the hippocampus, subsequently impairing the short-term spatial memory and learning capabilities. Administration of Tub A at a dose of 10 mg/kg resulted in a notable inhibition of HDAC6 expression, which in turn led to an increase in ChAT levels and restored the expression of tau protein, along with GSK3β phosphorylation [61]. These results support the notion that HDAC6 represents a viable therapeutic target for the treatment of AD.

#### 3.1.4 Stroke

HDAC6 significantly contributes to the pathogenesis of stroke by exacerbating neuroinflammation, oxidative stress, and axonal injury. HDAC6 induces excitotoxic injury and activates inflammatory pathways such as NF-κB. It also promotes apoptotic signaling by increasing p53 and caspase-3. Furthermore, HDAC6 activity is associated with BBB disruption and enhanced neuroinflammation through microglial activation and increased expression of inflammatory mediators, including iNOS, cyclooxygenase-2 (COX-2), and proinflammatory cytokines [62], [63]. Downregulation of HDAC6 with Tub A has been shown to protect neuronal integrity and attenuate inflammatory processes, demonstrating its potential as a neuroprotective agent in experimental stroke models [62]. In addition, the effects of Tub A (25 mg/kg and 40 mg/kg) were evaluated on a rat model of transient middle cerebral artery occlusion. The drug was administered via the i.p. route 24 h after the onset of ischemia, followed by daily doses for up to three consecutive days. Tub A promotes the activation of Akt, inhibits GSK3β, and consequently increases FGF-21 levels in the ischemic brain. This is achieved by reducing HDAC6 expression, suggesting that Tub A may offer a promising therapeutic approach for mitigating ischemic brain injury [29].

**Fig. 1:**
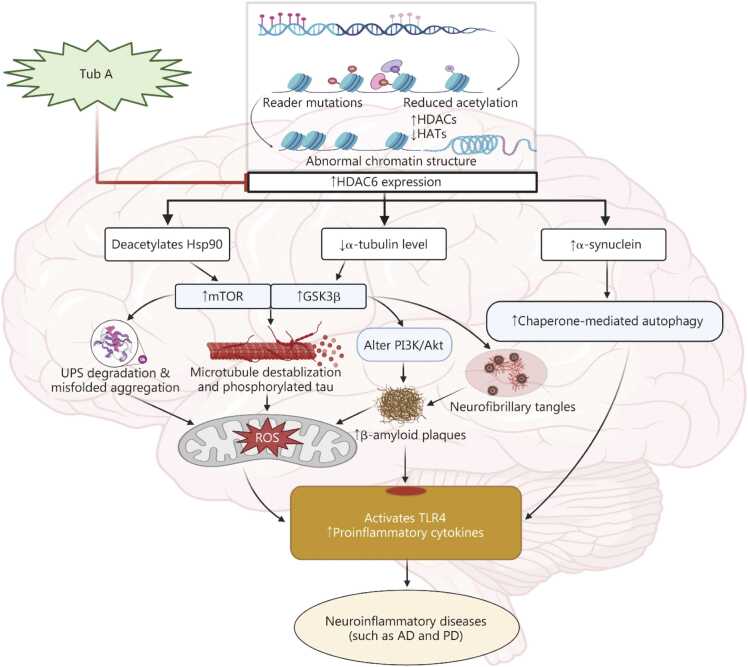
Effect of Tub A in neurodegenerative diseases. Tub A inhibited HDAC6 expression, leading to increased expression of α-tubulin, helping to restore the deacetylation of Hsp90 by inhibiting the mTOR/GSK3β signaling pathway. Ultimately, this process reduced misfolded protein aggregation and stabilized the disrupted microtubules. Tub A also modulated the PI3K/Akt pathway, reducing β-amyloid deposition and neurofibrillary tangles. Tub A decreased α-synuclein aggregation by reducing chaperone-mediated autophagy (CMA), ultimately reducing ROS levels. Additionally, Tub A led to a reduction in TLR4-mediated proinflammatory cytokine release, potentially targeting various types of neurodegenerative diseases. Collectively, these mechanistic actions underscore the therapeutic potential of Tub A in ameliorating neuroinflammatory and neurodegenerative conditions such as AD and PD. Tub A. Tubastatin A; HDAC6. Histone deacetylase 6; Hsp90. Heat shock protein 90; mTOR. Mammalian target of rapamycin; GSK3β. Glycogen synthase kinase 3β; PI3K/Akt. Phosphoinositide 3-kinase/protein kinase B; UPS. Ubiquitin-proteasome system; ROS. Reactive oxygen species; TLR4. Toll-like receptor 4; AD. Alzheimer’s disease; PD. Parkinson’s disease; HATs. Histone acetyltransferases.

Moreover, microglia are specialized cells derived from the myeloid lineage, located in the central nervous system, particularly in the brain and spinal cord. Their primary function is to maintain normal tissue homeostasis, which is essential for the overall health and stability of neural environments [64]. Microglia play a critical role in the severity of various diseases, as they can become activated or dysregulated [64]. One study revealed that LPS-induced inflammation in cultured rat microglia significantly reduced Sig1R expression. This effect was driven by inflammation resulting from TLR4 activation, which triggers the TLR4/TAK1/p38 MAPK signaling pathway and activates HDAC6. Importantly, pretreatment with Tub A at a concentration of 1 μmol/L significantly restored the *Sig1R* mRNA levels, targeting HDAC6 expression via regulation of the TAK1/p38 MAPK signaling pathway [51]. These findings indicate that Tub A shows significant potential as a targeted HDAC6 inhibitor for treating ischemic stroke and related neurological conditions, highlighting the need for further clinical investigations to evaluate its therapeutic efficacy **(**Fig. 1**)**.

### 3.2 Spinal cord injury

Spinal cord injury (SCI) is a condition that arises from trauma, medical illness, or degenerative disorders, resulting in a loss of function, sensation, and movement. The physical and neurological manifestations associated with SCI include paralysis, sensory loss, bowel and bladder dysfunction, muscle spasms, and chronic pain [65]. A study conducted by Wu *et al*. [66] found that elevated expression of HDAC6 influences microtubule stabilization, leading to the upregulation of microtubule-associated protein 1 light chain 3 beta (LC3B) and NLRP3. Additionally, HDAC6 promotes the secretion of inflammatory factors, which subsequently diminishes autophagy-lysosome transport and contributes to SCI. Treatment with Tub A at a dose of 50 mg/kg i.p. significantly inhibited the expression of HDAC6, further suppressing the inflammasome pathway and allowing microglial cells to phagocytize myelin debris. As a result, it influences the regulatory functions of the autophagy-lysosome pathway, presenting a novel potential therapeutic strategy for SCI [66].

Due to the high efflux ratio and limited BBB penetration of Tub A, treating spinal injuries remains challenging as higher doses and longer durations of administration are required. A study by Liao *et al*. [67] addressed this issue by developing a Tub A-loaded nanofibrous scaffold made of a poly(glycolide-co-ε-caprolactone) (PGCL)/silk fibroin (SF) matrix. This formulation allows for controlled, local drug release, enhancing the therapeutic efficacy of Tub A while minimizing its high-dose associated side effects [67]. The study provides substantial evidence about the therapeutic effect of Tub A in mitigating SCI. Future studies should focus more on exploring nanoformulation-based strategies to overcome these limitations.

### 3.3 Cardiovascular diseases

Cardiovascular diseases (CVDs) refer to conditions that affect the heart and circulatory system [68]. The prevalence of heart disease affects millions of patients globally. Recently, cardiovascular and circulatory diseases have emerged as the leading causes of death globally [69]. Among all CVDs, ischemic heart disease and cerebrovascular disease are the leading causes of mortality [70]. Environmental and lifestyle factors, including smoking, stress, dietary intake, health problems, and toxins, led to alterations in epigenetic mechanisms, which contribute to the development and progression of CVDs. Among all epigenetic changes, HDAC-mediated modifications play an important role in the progression of CVDs [71]. Numerous studies have also shown that reversible lysine acetylation and post-translational modifications are involved in the development of CVD risk conditions, including coronary artery disease, cardiac hypertrophy, heart failure (HF), myocardial infarction, and cardiomyopathy [72], [73]. In this context, HDAC6 has gained attention from researchers due to its ability to target tubulin, Hsp90, and cortactin, and stress-response pathways involved in cardiovascular pathology [40], [42].

Studies have shown that Tub A effectively regulates HDAC6 activity, impacting critical pathways associated with CVDs, including inflammation, fibrosis, and vascular remodelling. For example, Xu *et al*. [74] demonstrated that Tub A at a dose of 4.5 mg/kg i.p. alleviates post-resuscitation myocardial dysfunction by inhibiting HDAC6 activity, enhancing the acetylation and nuclear translocation of transcription factor EB (TFEB), involved in regulating gene expression related to autophagy, ultimately reducing the level of the NLRP3 inflammasome and cleaved caspase-1. Consequently, Tub A reduces cell apoptosis, leading to decreased levels of proinflammatory cytokines, improving myocardial function, and diminishing cardiac injury following cardiac arrest and resuscitation. *In vitro* studies using a hypoxia/reoxygenation (H/R) model showed that treatment with 40 μmol/L Tub A demonstrated cardioprotective effects by inhibiting NLRP3 inflammasome activation, reducing inflammatory signaling, and preventing apoptosis in H9c2 cardiomyocytes [74]. Similarly, Chi *et al*. [75] revealed that a dose of 50 mg/kg of Tub A via the i.p. route inhibited the expression of HDAC6, conferring anti-inflammatory, anti-oxidant, and cardioprotective effects. Additionally, the results of the *in vitro* study revealed that Tub A (5 μmol/L) treatment significantly inhibited the expression of HDAC6 in human aortic endothelial cells and HEK293 cells, leading to enhanced levels of acetylated cystathionine γ-lyase (CSEγ), inhibiting AngII-induced ubiquitination, protecting CSEγ from degradation, and upregulating CSEγ protein and hydrogen sulfide (H_2_S) levels, targeting AngII-induced vasoconstriction and hypertension, ultimately showing anti-inflammatory, anti-oxidant, and cardioprotective effects [75].

Pulmonary arterial hypertension (PAH) is a complex condition identified by elevated pulmonary vascular resistance, driven by a cancer-like, proliferative, and apoptosis-resistant phenotype of pulmonary artery smooth muscle and endothelial cells. As the disease progresses, it develops right ventricular failure and premature death. Boucherat *et al*. [76] demonstrated that administering a daily dose of Tub A at 25 mg/kg i.p. for two weeks significantly inhibited HDAC6 expression, increasing the acetylation of Ku70 at lysine residue 539, suppressing Bax, promoting mitochondrial membrane depolarization, and ultimately preventing apoptosis. As a result, there was improved right ventricular systolic pressure and mean pulmonary artery pressure in rats with Sugen-hypoxia [76]. Hence, Tub A represents a promising new target for treating PAH.

Increasing evidence suggests that inhibition of HDAC6 may represent a promising therapeutic strategy for cardiovascular complications, particularly those associated with ferroptosis, oxidative stress, and ischemia-induced cardiac injury. Song *et al*. [77] conducted a study demonstrating that 50 mg/kg of Tub A administered through the i.p. route daily for 4 weeks protects against doxorubicin-induced acute cardiomyopathy by inhibiting HDAC6 expression. A study by Leng *et al*. [78] evaluated the effect of Tub A in myocardial ischemia/reperfusion (MI/R) and H/R in diabetic rats and H9c2 cells, respectively. Tub A (10 mg/kg) intervention decreased cardiac infarction and ROS production and restored cardiac function with an upregulation of acetylated-Prdx1 levels in diabetic MI/R rats. Additionally, *in vitro*, findings revealed that a dose of 5 μmol/L of Tub A was found to have a protective effect against H/R injury in H9c2 cells. Furthermore, inhibition of increased HDAC6 activity in diabetic MI/R rats prevented oxidative stress by regulating the Prdx1 acetylation at K197. Overall, in both *in vivo* and *in vitro* studies, Tub A demonstrated cardioprotective effects [78].

Disruption of the neurohormonal axis contributes to increased ROS production and plays a central role in the pathogenesis of HF and arterial hypertension [79]. *In vitro* studies on H9c2 cardiomyocytes and adult mouse cardiomyocytes indicated that Tub A significantly inhibits HDAC6 expression, thereby reducing nicotinamide adenine dinucleotide phosphate hydrogen (NADPH) oxidase 2 (NOX-2) pathway activity. This reduction leads to decreased ROS production and increased telomere-specific anti-oxidant Prdx1 levels, resulting in a notable decrease in oxidative DNA damage. Additionally, it prevents telomere shortening in cardiomyocytes during HF and may open new therapeutic possibilities in the future [80]. Multiple research investigations demonstrated that increased NOX-2 pathway expression and decreased anti-oxidant Prdx1 levels play a crucial role in the development of cardiometabolic complications [81], [82], [83]. Inhibition of these pathways through Tub A significantly reduces cardiometabolic diseases, potentially paving the way for novel therapeutic strategies and possibly mitigating heart complications [84]. Tub A specifically inhibits HDAC6, reducing TNF-α and nicotinamide adenine dinucleotide production while enhancing myocardial mitochondrial complex I activity, thus reducing MI/R injury by preventing mitochondrial injury [26].

**Fig. 2:**
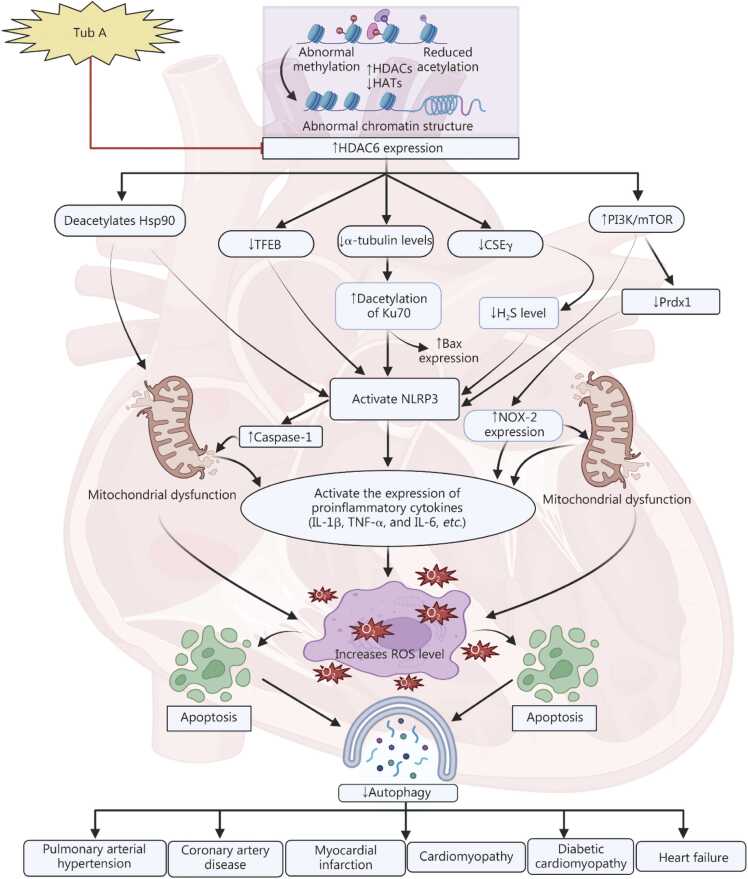
Effect of Tub A in cardiovascular diseases. Tub A exerts cardioprotective effects primarily through HDAC6 inhibition, leading to enhanced α-tubulin levels and mitigating the Hsp90 dysregulation. This modulation attenuates PI3K/mTOR signaling, promotes TFEB expression, Ku70 acetylation, and CSEγ upregulation, thereby suppressing Bax and caspase-1-mediated apoptosis linked to protecting mitochondrial dysfunction. Tub A also inhibits NLRP3 inflammasome activation and reduces proinflammatory cytokines, restoring mitochondrial function and autophagy. Increasing levels of Prdx1 while decreasing NOX-2 levels result in lower oxidative stress. Collectively, Tub A induced the autophagy process, potentially targeting cardiovascular disorders, including pulmonary arterial hypertension, coronary artery disease, myocardial infarction, cardiomyopathy, diabetic cardiomyopathy, and heart failure. Tub A. Tubastatin A; HDAC6. Histone deacetylase 6; TFEB. Transcription factor EB; Ku70. DNA repair protein Ku70; CSEγ. Cystathionine γ-lyase; H_2_S. Hydrogen sulfide; Prdx1. Peroxiredoxin 1; NOX-2. Nicotinamide adenine dinucleotide phosphate hydrogen oxidase 2; NLRP3. Nucleotide-binding oligomerization domain; leucine-rich repeat; and pyrin domain containing 3; IL-1β. Interleukin-1β; TNF-α. Tumor necrosis factor-α; IL-6. Interleukin-6; ROS. Reactive oxygen species; HATs. Histone acetyltransferases; mTOR. Mammalian target of rapamycin; Hsp90. Heat shock protein 90.

Overall, evidence suggests that alterations in epigenetic mechanisms, specifically increased HDAC6 expression, play a crucial role in the development of CVDs by affecting specific biological pathways. Tub A targets epigenetic modifications such as the overexpression of HDAC6, providing a new therapeutic option for disorders like myocardial infarction, HF, and hypertension. However, more research is needed to understand its molecular effects and assess its potential clinical use **(**Fig. 2**)**.

### 3.4 Cancer

Cancer is the second leading cause of death globally, following CVDs. Low- and middle-income countries account for a significant portion of global cancer mortality [85]. According to the World Health Organization (WHO), approximately 1 in 6 deaths worldwide is due to cancer [86]. In the United States, it is estimated that cancer will result in around 1700 deaths per day [87]. Studies have shown that epigenetic modifications play a significant role in the development and progression of cancers [88], [89]. Deacetylation, regulated by HDACs, is a critical process in cancer progression as it affects gene expression [90], [91]. Investigations indicated that overexpression of HDAC6 has been linked to an increased risk of various cancers, especially in advanced stages such as acute myeloid leukemia, breast cancer, oral squamous cell carcinoma, cutaneous T-cell lymphoma, ovarian cancer, and hepatocellular carcinoma [92]. Increased levels of HDAC6 have also been associated with enhanced migratory abilities in fibroblasts [93]. HDAC6 modulates multiple signaling pathways that regulate cellular proliferation and migration. Extracellular signal-regulated kinase (ERK) interacts and phosphorylates HDAC6, enhancing cell migration by promoting the deacetylation of α-tubulin. Additionally, ERK regulates Ras/MAPK/ERK, PI3K/Akt, and Wnt signaling pathways, which are involved in cellular proliferation and tumorigenesis [94], [95]. *In vivo* studies have shown that HDAC6 interacts with ERK1 and ERK2, key kinases in regulating cell growth and activation of epidermal growth factor receptor (EGFR) [96], [97]. Furthermore, through the deacetylation of Hsp90, HDAC6 also regulates the stability and functionality of Akt, impacting the PI3K/Akt signaling pathway, which is crucial for oncogenic processes such as cell survival, differentiation, migration, and angiogenesis [98]. This section focuses on the therapeutic potential of the HDAC6 inhibitor Tub A, which has demonstrated anti-tumor efficacy through the modulation of regulatory pathways [99].

Ferroptosis is a form of programmed cell death caused by iron-dependent lipid peroxidation and has emerged as a promising therapeutic mechanism against cancer. In cancer therapy, disruption of redox balance by the ionizing radiation (IR) induces ferroptosis and enhances the effectiveness of chemotherapy, indicating the importance of the ferroptosis mechanism in anti-cancer treatment [100], [101]. However, the production and clearance of iron-dependent lipid peroxides are tightly regulated by endogenous anti-oxidant enzymes, particularly glutathione peroxidase 4 (GPX4) [102]. GPX4 is the only enzyme in the GPX family that specifically reduces lipid peroxidation, thereby suppressing ferroptosis [103]. Studies have shown that inhibiting GPX4 activity increases ferroptosis via iron-dependent lipid peroxidation. For instance, a study by Liu *et al*. [32] demonstrated that Tub A inhibited GPX4 enzyme activity in a concentration- and time-dependent manner in both MDA-MB-231 and MCF-7 cells. In a mouse cancer model, treatment with Tub A at a dose of 2.5 mg/kg via subcutaneous (s.c*.*) route enhanced radiosensitivity by inhibiting HDAC6 and directly suppressing GPX4 activity. The compound also promoted radiotherapy-induced lipid peroxidation and tumor suppression in a xenograft model. These findings position Tub A as a promising ferroptosis inducer with potential as a therapeutic agent, alone or in combination with radiotherapy or immunotherapy. Besides ferroptosis induction, Tub A exhibited no significant toxic effects on the bone marrow, intestines, liver, kidneys, spleen, lungs, or heart [32]. Additionally, a study by Abouhish *et al*. [104] reported that Tub A exerts anti-oxidant effects by preserving nuclear factor erythroid 2-related factor 2 (Nrf2)-dependent gene expression and enhancing thioredoxin-1 activity. Hence, further evidence is needed to evaluate the effect of Tub A on oxidative parameters, focusing on GPX4 and Nrf2 regulation.

Cutaneous melanoma has become one of the fastest-growing tumors over the past four decades, with a notable increase in incidence among white populations [105]. Numerous studies have investigated how sun exposure affects the development of melanoma, confirming the influence of factors such as skin, hair, and eye pigmentation, as well as the presence of melanocytic nevi, through large-scale association analysis [106], [107]. Lifestyle factors and medical exposures, including the use of immunosuppressive drugs and nonsteroidal anti-inflammatory medications, have also been linked to an increased risk of melanoma [108], [109]. Additionally, a genome-wide association study has identified genetic loci linked to known risk factors, highlighting genetic susceptibility to melanoma [110]. A study has shown that the genetic and pharmacological inhibition of HDAC6 by Tub A reduces melanoma cell proliferation in both *in vitro* and *in vivo* models [111]. HDAC6 has been identified as a regulator of immune-related pathways in melanoma, including its role in programmed death receptor ligand 1 (PD-L1) regulation. PD-L1 is a protein whose overexpression is often associated with poor prognosis in several cancers, including melanoma [112], [113]. HDAC6 also modulates the signal transducer and activator of transcription 3 (STAT3) pathway, which is frequently altered in melanoma and other malignancies. Lienlaf *et al*. [114] demonstrated that selective HDAC6 inhibitors, such as Tub A, hinder tumor cell survival and enhance immune recognition, exhibiting notable anti-tumor activity, particularly in melanoma models.

Breast cancer is the most common malignant disease affecting women, with its development influenced by various genetic and epigenetic factors [115]. Epigenetic modifications, particularly those involving HDAC6, play a significant role in breast cancer progression, highlighting the therapeutic potential of targeting HDAC6 [116], [117]. A study found that treating MDA-MB-231 human breast cancer cells with Tub A or palladium nanoparticles (PdNPs) resulted in a dose-dependent decrease in cell viability [118]. Tub A effectively inhibited HDAC6 expression and, when combined with PdNPs, modulated the expression of key apoptosis-related proteins, including p53, Bax, Bak, Bcl-2, caspase-3, and caspase-9. This combination treatment promoted apoptosis and exhibited a synergistic effect [118].

Moreover, glioblastoma multiforme (GBM) often linked to the loss of primary cilia, remains one of the most challenging cancers to treat due to its heterogeneous nature and nonspecific clinical symptoms, such as seizures and headaches [119]. Urdiciain *et al*. [120] demonstrated that when used alone, Tub A increased acetylated α-tubulin levels and potentially reduced the mRNA expression of *GLI1* and *PTCH1*, downregulating the sonic hedgehog (Hh) pathway. Additionally, Tub A inhibited HDAC6 expression, which mitigates TGF-β-induced downregulation of E-cadherin, thereby reversing epithelial-mesenchymal transition (EMT) and promoting apoptosis in GBM cells, particularly in T98G and LN405 cell lines [120]. Meanwhile, Pezzotta *et al*. [121] investigated the effects of Tub A using a concentration of 8 μmol/L for treating the GBM using the U87-MG human GBM cell line and 25 μmol/L for treating a zebrafish embryo model. Their findings showed that combined inhibition of HDAC6 and the Hh pathway disrupted lysosomal degradation and autophagic flux, resulting in the accumulation of metabolic and autophagic substrates and reduced GBM cell viability [121]. Furthermore, Zhang *et al*. [122] demonstrated that a 20 µmol/L dose of Tub A, in combination with a COX-2 inhibitor, synergistically enhanced anti-tumor activity in CAL 27 and SACC-83 cells. This combination treatment activated phosphatase and tensin homolog (PTEN) and inactivated Akt [122]. Similarly, neural precursor cell expressed, developmentally downregulated 9 (NEDD9), a scaffolding protein, is upregulated in various cancers, including ovarian, glioblastoma, melanoma, and breast carcinomas. NEDD9 activates Aurora A kinase (AURKA), which induces HDAC6 phosphorylation, thereby increasing its deacetylase activity [123]. Kozyreva *et al*. [123] demonstrated that Tub A significantly reduced pulmonary metastases by inhibiting AURKA and HDAC6 activity in breast cancer xenografts.

**Fig. 3:**
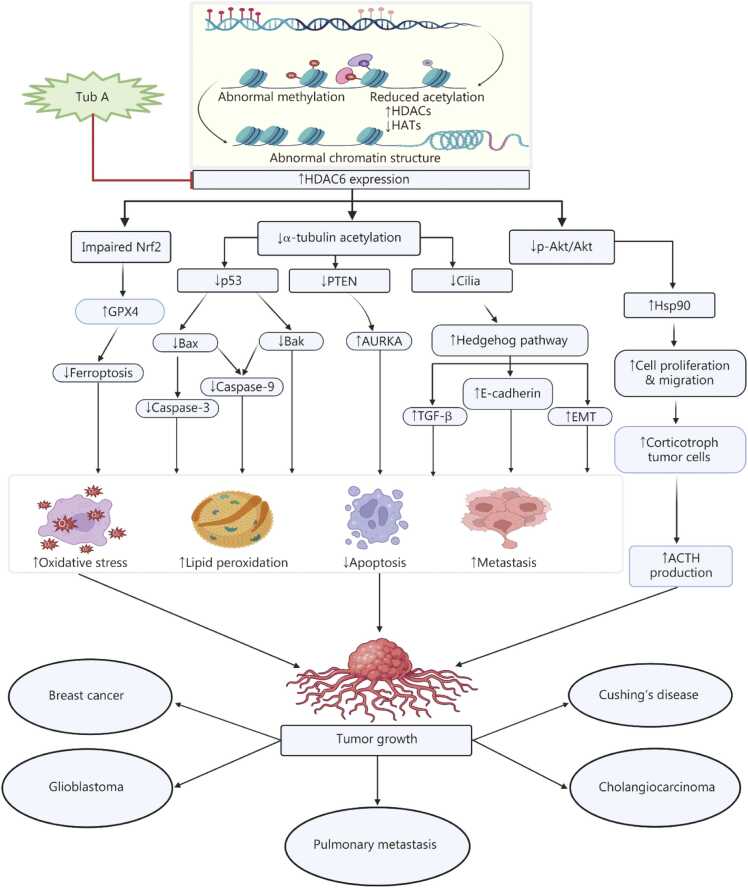
Effect of Tub A in several types of cancer. Tub A inhibits HDAC6 overexpression, thereby restoring histone acetyltransferase enzyme and providing a loose chromatin structure that activates gene expression. Furthermore, it promotes increased acetylation of α-tubulin, upregulates the expression of Nrf2, and modulates the Akt signaling pathway. Moreover, Tub A treatment increased acetylation of α-tubulin, leading to restored p53 activity and increased activation of Bax, Bak, and caspase-3 & -9, ultimately reducing lipid peroxidation. Tub A increases PTEN activity, inhibits the PI3K/Akt signaling, reduces AURKA stability and expression, leading to cell cycle arrest, impaired mitotic spindle formation, and increased cancer cell apoptosis, which reduces tumor proliferation. Tub A reduces GPX4 expression, inducing ferroptosis, which decreases oxidative stress and activates apoptosis and autophagy, thereby enhancing anticancer efficacy. Tub A enhances α-tubulin acetylation and improves cilia function, which in turn suppresses the abnormal activation of the hedgehog pathway. This suppression results in decreased levels of TGF-β, E-cadherin, and EMT activity, ultimately contributing to reduced proliferation and metastasis in cancer cells. Also, Tub A increases Hsp90 acetylation**,** impairs its chaperone function**,** and reduces cell proliferation and migration, playing a role in reduced tumour progression via decreased ACTH production. Based on these mechanisms, Tub A demonstrates therapeutic potential in various types of cancer, such as breast cancer, glioblastoma, and cholangiocarcinoma, as well as in conditions such as pulmonary metastasis and Cushing’s disease. Tub A. Tubastatin A; HDAC6. Histone deacetylase 6; HATs. Histone acetyltransferases; Nrf2. Nuclear factor erythroid 2-related factor 2; GPX4. Glutathione peroxidase 4; p53/PTEN. Phosphatase and tensin homolog tumor suppressor proteins; AURKA. Aurora kinase A; TGF-β. Transforming growth factor-β; EMT. Epithelial-mesenchymal transition; Hsp90. Heat shock protein 90; ACTH. Adrenocorticotropic hormone; ROS. Reactive oxygen species.

Based on a comprehensive review of research investigations, Tub A potentially inhibits the expression of HDAC6, thereby influencing multiple cancer-related pathways and targeting epigenetic mechanisms (Table 1 [29], [32], [47], [50], [51], [53], [54], [111], [118], [120], [121], [122], [123] **and** Table 2 [29], [32], [44], [46], [47], [48], [49], [53], [57], [60], [61], [66], [111], [114], [121], [122], [123]). However, further research is necessary to explore the molecular pathways involved. Interestingly, clinical trials should be prioritized to assess the drug’s potential as a targeted therapy for cancer treatment **(**Fig. 3**)***.*

**Table 1:** *In vitro* studies on neurodegenerative and cancer diseases.

**Diseases**	**Model and cell lines**	**Dose of Tub A**	**Mechanism**	**Inference**	**References**
Neurodegenerative diseases					
Parkinson’s disease (PD)	6-OHDA-model in SH-SY5Y cells	15 µmol/L	Inhibited the deacetylation activity of HDAC6, provided an anti-apoptotic effect, reversed the NLRP3 inflammatory response, and protected dopaminergic neurons	These findings suggest that Tub A inhibits the deacetylase catalytic domain of HDAC6, a potential target for PD	[47]
Proteasomal degradation	HEK293T and PC12 cells co-transfected with Flag-XBP1s and HA-Ub plasmids	3 µmol/L	Inhibits the HDAC6, increases XBP1 protein expression, and enhances the expression of anti-oxidative genes	Tub A decreased the expression of oxidative genes, which therapeutically target the acetylation-mediated process of proteasomal degradation	[50]
Neuroinflammation	30 or 100 ng/ml LPS induced inflammation in rat primary cultured microglia	1 μmol/L	Inhibits HDAC6 expression, reduces TLR4 level, and enhances *Sig1R* mRNA levels via regulation of TAK1/p38 MAPK pathway	These findings indicate that Tub A potentially mitigates ischemic stroke-related neurological disorders	[51]
Giant axonal neuropathy (GAN)	Cultured embryonic DRG neurons derived from *Gan*^−/−^ mice and HEK293 cells	1 µmol/L	Inhibiting HDAC6 activity increases tubulin acetylation, facilitating the restoration of axonal transport of organelles in cultured neurons	Tub A inhibits histone deacetylase activity, enhancing axonal transport, and is considered a potential treatment for GAN disease	[53]
Glioblastoma multiforme (GBM)	Human glioblastoma cell lines (U343, U373, U138, LNZ308, A172, U118, U251, and U87)	32 µmol/L	Tub A regulates the p97/VCP-facilitated UPS degradation pathway by inhibiting HDAC6 overexpression, and reduces the clearance of aggregated, ubiquitinated proteins	The research findings suggest that targeting HDAC6 expression with Tub A is an effective therapeutic strategy against glioma	[54]
Stroke-induced brain infarction	Glutamate-treated rat primary cortical neurons	0.1, 0.25, 0.5, 0.75, and 1 μmol/L	Inhibits HDAC6 expression, enhances acetylated α-tubulin, and restores mitochondrial trafficking in rat cortical neurons exposed to glutamate-induced excitotoxicity, providing neuroprotection	Tub A inhibits the activity of HDAC6, which is a promising target for treating ischemic stroke	[29]
Cancers					
Breast cancer	Human breast cancer cell lines MDA-MB-231 and MCF-7 cells	0 to 8 μmol/L	Induces ferroptosis by directly inhibiting GPX4 enzymatic activity	Tub A demonstrates anti-tumor effects when used alongside radiotherapy	[32]
Melanoma tumor growth	Melanoma cell lines (B16-F10 luc murine melanoma cell line)	3 μmol/L	Inhibits HDAC6 activity, decreases cell proliferation, and causes G1 arrest without inducing apoptosis	Tub A could be a potential therapeutic agent for melanoma cancer via targeting HDAC6	[111]
Human breast cancer	MDA-MB-231 human breast cancer cells	4 μmol/L	Inhibits HDAC6 expression, and when combined with PdNPs, it modulates the expression of key proteins involved in apoptosis, such as p53, Bax, Bak, Bcl-2, caspase-3, and caspase-9, and promotes apoptosis	The overall findings revealed that the therapeutic efficacy of the Tub A and PdNPs combination suggests that it could be a potential targeted therapy for breast cancer	[118]
Glioblastoma	T98G and LN405 glioblastoma cell lines	Tub A dose in the LN405 cell is 27.5−32.5 µmol/L and in T98G cells is 30 µmol/L	Inhibits HDAC6 expression, increases acetylated α-tubulin levels, and reduces mRNA expression of *GLI1* and *PTCH1*, downregulating the Hh pathway, further mitigating TGF-β-induced downregulation of E-cadherin, and promotes apoptosis	The results demonstrate that treatment with Tub A significantly reduced glioblastoma cells’ clonogenicity and migratory capacity	[120]
GBM	U87-MG cells	8 μmol/L	Inhibiting HDAC6 and the Hh pathway disrupts proper lysosomal degradation and promotes the accumulation of metabolic and autophagic substrates	Tub A treatment reduced the growth of glioblastoma tumours in U87-MG cells	[121]
Human tongue squamous cell carcinoma, human salivary adenoid cystic cancer, and human malignant glioblastoma cells	CAL 27, SACC-83, and U-87 MG cells	20 µmol/L	It inhibits HDAC6, upregulates membrane-bound PTEN, downregulates phospho-Akt, induces apoptosis, and inhibits proliferation	This study demonstrated that combining an HDAC6 inhibitor (Tub A) and a COX-2 inhibitor produces synergistic antitumor effects	[122]
Pulmonary metastases	MDA-MB-231, MDA-231-LN, BT549, and HEK293T cells transfected with plasmid DNA and/or siRNA using nucleofection	10−20 μmol/L	Inhibits the expression of HDAC6, reduces the expression of NEDD9, and also inhibits the expression of AURKA	Tub A, or combined with MLN8237, effectively reduced pulmonary metastases	[123]

HDAC6. Histone deacetylase 6; 6-OHDA. 6-hydroxydopamine; NLRP3. Nucleotide-binding oligomerization domain, leucine-rich repeat, and pyrin domain containing 3; XBP1. X-box binding protein 1; LPS. Lipopolysaccharide; Tub A. Tubastatin A; Sig1R. Sigma-1 receptor; TLR4. Toll-like receptor 4; MAPK. Mitogen-activated protein kinase; TAK1. Transforming growth factor-β-activated kinase 1; DRG. Dorsal root ganglia; GPX4. Glutathione peroxidase 4; PdNPs. Palladium nanoparticles; GLI1. Glioma-associated oncogene homolog 1; PTCH1. Patched homolog 1; TGF-β. Transforming growth factor-β; PTEN. Phosphatase and tensin homolog; NEDD9. Neural precursor cell expressed; developmentally down-regulated 9; AURKA. Aurora kinase A; COX-2. Cyclooxygenase-2; Hh. Sonic hedgehog; UPS. Ubiquitin-proteasome system

**Table 2:** *In vivo* studies on neurodegenerative and cancer diseases.

**Diseases**	**Model**	**Dose and route of Tub A**	**Mechanism**	**Inference**	**References**
Parkinson’s disease (PD)	6 μg of 6-OHDA-in C57BL/6 mice by stereotaxic injection	25 mg/kg i.p.	Inhibits the expression of HDAC6, resulting in enhanced acetylation of Prdx1 and Prdx2, which ultimately reduces the production of reactive oxygen species, increases anti-oxidant activity, and reduces oxidative stress	Research findings suggest that inhibiting the HDAC6 activity through Tub A reduces dopaminergic injury and could mitigate PD	[46]
	2 μl of human wild-type α-synuclein AAV2 virus in Wistar rats	15 mg/kg i.p.	Inhibits the expression of HDAC6, protecting dopaminergic neurons in the substantia nigra of rats. This leads to the upregulation of key components of chaperone-mediated autophagy, increased protein acetylation, and reduced neuroinflammation	The findings indicate that Tub A ameliorates PD in a rat model	[44]
	500 µmol/L MPP^+^ -induced PD model in Zebrafish	1 µmol/L	Inhibits the deacetylation of tubulin K40, inhibits the HDAC6 activity, enhances anti-oxidative genes, and protects against oxidative stress	This study indicates that inhibiting HDAC6 isoforms in zebrafish can effectively restore cellular metabolism in the PD model	[49]
	PD model was induced through stereotaxic injection of 9 μg of 6-OHDA in C57BL/6 mice	25 mg/kg i.p.	Inhibiting the expression of the HDAC6 enzyme reduces the levels of NLRP3, IL-1β, and mature caspase-1, decreasing oxidative stress and increasing Prdx2 acetylation levels	Pharmacological inhibition of HDAC6 using Tub A reduces NLRP3 inflammation and protects dopaminergic neurons, highlighting its potential to mitigate PD	[47]
	Stereotaxic microinjection of lactacystin induced nigrostriatal dopamine neuron degeneration in C57BL/6 mice	25 μg/g i.p.	Inhibition of HDAC6 results in increased acetylated α-tubulin levels and modulates protective responses to cytotoxic α-synuclein aggregates	This study revealed that Tub A improved behaviour deficits in PD	[48]
Giant axonal neuropathy (GAN)	Gan^−/−^ mice with overexpression of the peripherin (Prph) transgene	25 mg/kg i.p.	Inhibits the overexpression of HDAC6, leading to increased tubulin acetylation levels and reduced abnormal accumulation of Prph and neurofilament proteins	Tub A has the potential to treat GAN	[53]
Alzheimer’s disease (AD)	AβPPswe/PS1^Δ^E9 (PAP) double-transgenic mice C57BL/6 J	50 mg/kg i.p.	Inhibits HDAC6 activity, mitigates tau pathology by reducing tau phosphorylation and enhancing autophagy-related degradation through the Akt/GSK3β/mTOR signaling pathway	Findings indicate that selective inhibition of HDAC6 by Tub A/ACY-1215 ameliorates cognitive impairment in the AD mouse model	[60]
	rTg4510 mouse models of tau deposition and non-transgenic mice	25 mg/kg i.p.	Inhibits the overexpression of HDAC6 and enhances the acetylation level of α-tubulin, increases acetylation of Hsp90, ultimately reduces total tau levels, and consequently improves behavioural deficits	The findings demonstrated that Tub A shows effective therapeutic strategies for AD and other tauopathies	[57]
Ischemic brain injury	Rat model of transient MCAO	25 mg/kg and 40 mg/kg i.p.	Inhibits the expression of HDAC6, promotes the activation of Akt, inhibits GSK3β, and consequently upregulates acetylated α-tubulin, and increases FGF-21 levels	Tub A intervention could be a promising therapeutic approach for mitigating ischemic brain injury	[29]
Brain injury	Injected 1 µl APOE4+1 µl Aβ_1-40_ via surgery in Sprague Dawley rats	10 mg/kg injected surgery	Inhibits HDAC6 expression, increases mRNA expression of *ChAT 1*, restores the expression of tau protein, along with GSK3β phosphorylation, and decreases the short-term spatial memory and learning ability	The findings suggest that Tub A mitigates brain injury induced by APOE4 and Aβ_1-40_ coaggregation in the rat model	[61]
Spinal cord injury (SCI)	Surgery of the T10 spinal cord in C57BL/6 mice	50 mg/kg i.p.	Inhibits the expression of HDAC6, reduces the secretion of inflammatory factors, increases the IgG levels, decreases the IgM levels, and enhances MEC-mediated myelin debris clearance	The inhibition of HDAC6 by Tub A regulates immune-inflammatory responses, thereby presenting a novel therapeutic strategy for of SCI	[66]
Cancer	2×10^6^ MDA-MB-231 cells were injected into mice via s.c. route	2.5 mg/kg s.c. route	Enhances radiosensitivity by inhibiting HDAC6, directly suppressing GPX4 activity and reduced tumor.	These findings highlight Tub A use alone or with radiotherapy/immunotherapy potentially targets cancer	[32]
Melanoma tumor	Tumour induced in C57BL/6 mice via the s.c. route with B16-F10-luc wild type cells	25 mg/kg s.c.	Inhibits the activity of HDAC6 and reduces melanoma cell proliferation	A combination of Tub A exhibits significant anti-tumour activity, particularly in melanoma models, but the context dependency of HDAC inhibition needs to be further investigated	[111]
	1.3×10^5^ B16‐F10 melanoma cells injected via the s.c. route in mice	25 mg/kg i.p.	Inhibits HDAC6 activity, modulates tumour cell survival and enhances immune recognition, and downregulates PD-L1, PD‐L2, B7‐H4 and TRAIL‐R1 expression	Results show that Tub A exhibits anti-tumour effects in melanoma cancer	[114]
Glioblastoma multiforme	Zebrafish model	25 µmol/L	Inhibits HDAC6 expression, targets the hedgehog pathway, which impairs lysosomal degradation, and promotes the accumulation of metabolic and autophagic substrates	Tub A reduces the growth of glioblastoma in the zebrafish embryo model	[121]
Tongue squamous cell carcinoma	CAL 27 cells injected via s.c. route, inoculated (5×10^6^ cells/mouse) in BALB/c nude mice	0.5 mg/kg i.p.	Inhibits the HDAC6 expression, activates PTEN, and dephosphorylates Akt	Tub A, in combination with a COX-2 inhibitor, synergistically induced an anti-tumour effect	[122]
Pulmonary metastases	Human MDA-MB-231 LN 1×10^5^ tumour cells injected via i.v. in NOD-Scid-gamma female mice	40 mg/kg i.p.	Inhibits the AURKA and HDAC6 activity, and decreases the pulmonary metastases	Tub A, when combined with other treatments, shows therapeutic effects in xenograft models of breast cancer and reduces pulmonary metastases	[123]

HDAC. Histone deacetylase; 6-OHDA. 6-hydroxydopamine; NLRP3. Nucleotide-binding oligomerization domain, leucine-rich repeat, and pyrin domain containing 3; Tub A. Tubastatin A; GPX4. Glutathione peroxidase 4; Prdx. Peroxiredoxin; IL-1β. Interleukin-1β; GSK3β. Glycogen synthase kinase 3β; mTOR. Mechanistic target of rapamycin; Hsp90. Heat shock protein 90; FGF-21. Fibroblast growth factor 21; ChAT 1. Choline acetyltransferase 1; APOE4. Apolipoprotein E4; Aβ_1-40_. Amyloid beta 1–40 peptide; s.c. Subcutaneous; i.p. Intraperitoneal; MCAO. Middle cerebral artery occlusion; PTEN. Phosphatase and tensin homolog; PD-L1. Programmed death receptor ligand 1

### 3.5 Role of Tubastatin A in metabolic, muscular dystrophy, and inflammatory conditions

#### 3.5.1 Metabolic disorders

Metabolic disorders are expected to become the most common non-communicable diseases affecting around one billion people in the coming decades [124]. High fasting blood sugar, low high-density lipoprotein cholesterol, high blood pressure, obesity, and abnormal liver enzyme levels are all indicators of metabolic syndrome. This condition greatly increases the risk of diabetes and CVDs, along with other chronic health issues that can lead to premature death and increased overall mortality. It has become one of the most pressing health issues of the current era [125]. Several studies have demonstrated that alterations in epigenetic mechanisms cause a wide range of disorders, including metabolic disorders [126], [127]. The epigenetic alterations affect the metabolic system by causing hypermethylation and deacetylation of many tissue-specific clock genes, which in turn lead to elevated cholesterol levels, impaired glucose tolerance and insulin resistance, obesity, and altered gut microbiome homeostasis [128], [129], [130]. Targeting aberrant epigenetic mechanisms, precisely, dysregulated HDAC6 activity can result in a more targeted and effective therapeutic approach for metabolic and age-related disorders [43], [131], [132], [133], [134].

It has been reported that increased expression of HDAC6 triggers TLR4 production, leading to an increase in proinflammatory genes such as *IL-1β*, *IL-6*, and *TNF-α* via ROS-MAPK-NF-κB/AP-1 pathway in macrophages, as well as an increase in expression of NADPH oxidase. HDAC6 overexpression reduces the level of acetylated α-tubulin, which enhances macrophage expression [21], [135]. Additionally, a study demonstrated that elevated HDAC6 expression leads to increased TLR4 expression in acute liver failure (ALF), and TLR4-mediated MAPK and NF-κB signaling pathways are involved in inflammation and ALF diseases [136]. Furthermore, Zhang *et al*. [137] reported that HDAC6 interacts with FOXO1, promoting autophagy and lipogenesis, accelerating the accumulation of lipids and causing liver steatosis. Obesity development has also been associated with hypothalamic dysfunction. The pathophysiology of obesity and diabetes has been linked to impairments in proteostatic processes such as autophagy, the heat shock response, the ubiquitin-proteasome pathway, and integrated stress responses [138]. Notably, HDAC6 regulates several of the above-mentioned pathological processes. A study showed that Tub A selectively inhibits HDAC6 expression, which is associated with reduced weight gain in mice fed a high-fat diet [27].

#### 3.5.2 Duchenne muscular dystrophy

HDAC6 has emerged as a potential therapeutic target in duchenne muscular dystrophy (DMD) due to its role in regulating cytoskeletal dynamics, oxidative stress, and protein homeostasis in skeletal muscle. Osseni *et al*. [139] demonstrated that Tub A, administered at 25 mg/(kg·d) via the i.p. route in the mdx mouse model of DMD, an inherited condition characterized by progressive muscle fibre degeneration, selectively inhibited HDAC6 expression. *In vitro*, a 5 µmol/L dose of Tub A treated C2C12 cells, downregulating TGF-β signaling through enhanced Smad3 acetylation. This inhibition reduced the phosphorylation, nuclear translocation, and transcriptional activity of Smad2 and Smad3, leading to decreased expression of atrogenes (*MAFbx* and *MuRF1*) and activation of the mTOR pathway. These findings suggest Tub A could be a potential drug candidate for treating DMD [139]. Further investigation demonstrated that Tub A (70 mg/kg) enhances the acetylation of Prdx, protecting it from hyperoxidation and modulating the intracellular redox status in mdx mice by significantly inhibiting HDAC6 expression. It downregulates nicotinamide adenine dinucleotide phosphate hydrogen oxidase 2 (NOX-2) activity in mdx mice and promotes autophagosome maturation, enhancing autophagy through tubulin acetylation. These findings suggest that HDAC6 inhibition by Tub A can improve protein homeostasis and redox regulatory mechanisms associated with dystrophic phenotypes and improve muscle function [140]. However, further studies with comprehensive molecular evaluations are required to establish Tub A’s therapeutic efficacy against DMD prior to clinical use.

#### 3.5.3 Colitis

HDAC6-mediated inflammatory signaling plays an important role in the development of inflammatory bowel diseases, suggesting that targeting HDAC6 could be a potential therapeutic approach for colitis. However, the clinical use of Tub A is challenging due to its rapid clearance, which limits its ability to reach and maintain sufficient concentrations at the inflamed intestinal tissue. A study showed that Tub A-thioketal nanoparticles (TKNP) exhibited potential anti-inflammatory effects in both *in vitro* and *in vivo* models of dextran sodium sulfate-induced colitis mice. Due to the high clearance rate of Tub A, it was loaded in ROS-responsive TKNP, a nanoparticle that releases the entrapped drug at the site of a high ROS environment, resulting in a higher local concentration of loaded drug. It was found that, in the presence of ROS environment, the release of Tub A from TKNP was around 70%, compared to only 8% in the absence of ROS [141]. Increased concentration of Tub A in inflamed colon tissue enhanced HDAC6 suppression, leading to a decline in proinflammatory cytokine levels. In addition, HDAC6 inhibition enhanced microtubule acetylation and release of IL-10, an anti-inflammatory cytokine [141]. These findings highlight the potential of ROS-responsive TKNP for localized delivery of Tub A and support further preclinical evaluation as a therapeutic strategy for ulcerative colitis.

#### 3.5.4 Hepatic complications

LPS-induced inflammation is a widely used model to investigate inflammation caused by infectious and inflammatory conditions [142]. Wang *et al*. [143] findings demonstrated that in LPS-stimulated primary bovine mammary epithelial cells, overexpression of HDAC6 significantly enhanced the production of ROS by upregulating NADPH oxidase activity and modulating inflammatory cytokine levels. In contrast, treatment with Tub A markedly reduced the release of proinflammatory cytokines, including TNF-α and IL-1β. This effect was accompanied by increased acetylation of α-tubulin, which modulated the ROS/NF-κB signaling pathway [143]. These results indicate that overexpressed HDAC6 increases oxidative stress and inflammatory signaling and suggest that its inhibition may attenuate LPS-induced inflammatory response. In addition to HDAC6 involvement in inflammatory signaling, HDAC6 also regulate microtubule dynamics through the primary cilium, a cell surface organelle critical for sensing extracellular signals, which plays a vital role in ciliary-EGFR signaling. Its dysfunction has been associated with chronic liver diseases, including polycystic liver disease and cholangiocarcinoma [144]. In a study by Pant *et al*. [144], the findings indicate that, in ciliary-defective cholangiocytes, EGFR remains on the surface of cells and becomes overstimulated. However, inhibition of HDAC6 by Tub A restored cilia by targeting EGFR and ERK phosphorylation. In an orthotopic rat model, this inhibition led to EGFR translocation to primary cilia [144]. This restoration of ciliary function may offer a therapeutic approach for EGFR-driven diseases such as polycystic liver disease and cholangiocarcinoma.

Ischemia-reperfusion injury is a significant cause of morbidity following liver transplantation and other medical conditions or surgeries, occurring in approximately 15% of liver transplant recipients. It leads to early allograft dysfunction, increased allograft loss, and poorer long-term outcomes [145], [146]. The findings suggest that Tub A-mediated protection targets the ubiquitin/aggresome/inflammasome pathways through direct inhibition of HDAC6, offering a potential therapeutic approach for hepatic ischemic injury [147].

**Fig. 4:**
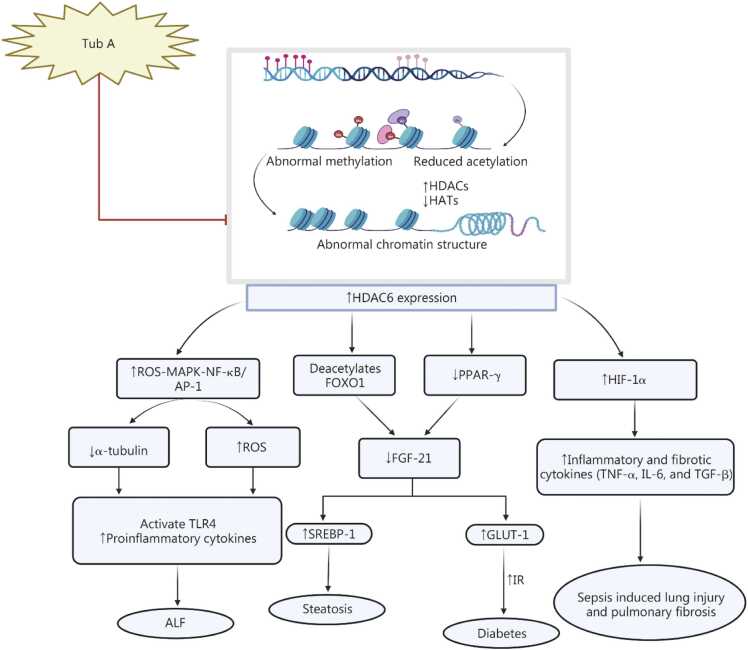
Effect of Tub A in metabolic diseases and sepsis-induced lung injury. Tub A inhibits the expression of HDAC6 through the enhancement of acetylation of the HAT enzyme. This mechanism reduces the ROS-MAPK-NF-κB/AP-1 signaling pathway, which ultimately contributes to increased levels of α-tubulin. Consequently, this process leads to decreased ROS production and reduced TLR4-mediated release of proinflammatory cytokines. These combined effects serve to attenuate the severity of ALF. Tub A also deacetylates FOXO1 expression while upregulating expression of PPAR-γ and FGF-21, contributing to reduced hepatic steatosis through suppression of SREBP-1 and improved insulin sensitivity via downregulation of GLUT-1, ultimately, mitigating the diabetic condition. Moreover, Tub A lowers HIF-1α expression, thereby diminishing inflammatory cytokine levels like TNF-α and IL-6, targeting sepsis. The mechanisms described emphasize the therapeutic potential of Tub A in mitigating metabolic disorders and sepsis conditions. Tub A. Tubastatin A; HDAC6. Histone deacetylase 6; HATs. Histone acetyltransferases; MAPK. Mitogen-activated protein kinase; NF-κB/AP-1. Nuclear factor κB/activator protein 1; TLR4. Toll-like receptor 4; FXOX1. Forkhead box O1; PPAR-γ. Peroxisome proliferator-activated receptor gamma; FGF-21. Fibroblast growth factor 21; SREBP-1. Sterol regulatory element-binding protein 1; GLUT-1. Glucose transporter type 1; IR. Insulin resistance; HIF-1α. Hypoxia-inducible factor-1α; TNF-α. Tumor necrosis factor-α; IL-6. Interleukin-6; ALF. Acute liver failure; ROS. Reactive oxygen species.

The current section explains that Tub A specifically targets HDAC6 activity, influencing various pathways that can help alleviate metabolic diseases, DMD, colitis, and other hepatic complications. However, additional preclinical research is needed to explore how Tub A affects other metabolic pathways and to identify its potential targets in diseases. As mentioned earlier, this can be achieved through molecular studies. We suggest that Tub A could play a therapeutic role in clinical studies, particularly given its minimal adverse effects **(**Fig. 4**)**.

### 3.6 Autoimmune diseases

The global prevalence of autoimmune diseases contributes to increased morbidity and mortality rates [148]. Autoimmune diseases result from immune system dysregulation and lead to excessive production of autoantibodies, causing immune-mediated damage to healthy organs and tissues [148]. Autoimmune diseases have a complex pathogenesis involving multiple factors that disrupt autoimmune processes and immunological tolerance. These factors include environmental, genetic, psychological, and infectious factors, although some causes may remain unidentified [149]. Various epigenetic mechanisms, including DNA methylation and histone modification, play crucial roles in the development of autoimmune diseases. Additionally, abnormal epigenetic mechanisms offer valuable insights into autoimmune disease pathogenesis and progression [150]. Hence, new therapeutic strategies are urgently needed to prevent and treat this chronic condition.

Cell migration, modulation of the immunological response, viral infection pathways, and the degradation of misfolded proteins are among the critical biological processes associated with the regulatory functions of HDAC6 [16]. Research has demonstrated that targeting HDAC6 to induce epigenetic modifications holds significant therapeutic potential [31]. This insight has accelerated the development of epigenetic therapies as a potential strategy for treating autoimmune diseases. Oh et al. [151] compared CKD-L (another HDAC6 inhibitor, 15 or 30 mg/kg via s.c. route) and Tub A (30 mg/kg via s.c. route), in a model of collagen-induced arthritis in DBA/1 J mice (bovine type II collagen). Their results revealed that both CKD-L and Tub A significantly inhibited HDAC6 activity, which led to a reduction in synovial inflammation and a decrease in the production of proinflammatory cytokines. However, CKD-L has better therapeutic potential to treat rheumatoid arthritis as compared to Tub A [151]. Another study found that Tub A, along with largazole, was used to mitigate arthritis. The treatment promisingly reduced synovial inflammation and protected the joints. The expression of vascular cell adhesion molecule (VCAM)-1 and intracellular adhesion molecule (ICAM)-1, induced by TNF-α, was reduced by Tub A, added at 10 μmol/L along with largazole [152]. Vishwakarma *et al*. [153] conducted an *in vitro* study that showed the Tub A treatment inhibited the release of TNF-α and IL-6 in LPS-stimulated RAW264.7 and THP-1 cells in a dose-dependent manner. *In vivo* study results indicated Tub A (30 mg/kg) significantly reduced paw edema induced by the intraplantar administration of Freund’s complete adjuvant in animal models. These observations are consistent with an anti-inflammatory effect of Tub A, mediated through HDAC6 inhibition and subsequent reduction of TNF-α, IL-6, and nitric oxide production [153].

**Fig. 5:**
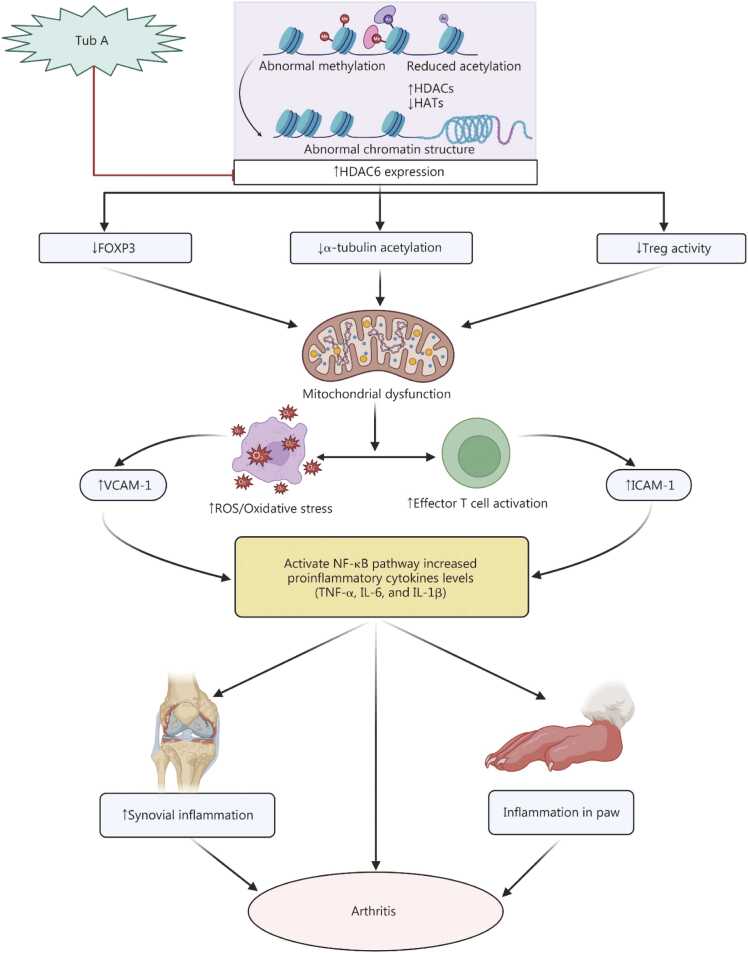
Effect of Tub A on autoimmune disease. Tub A increases acetylation of α-tubulin, linked to enhanced expression of FOXP3 and improved Treg activity, ultimately protecting against mitochondrial dysfunction. This results in a reduction in oxidative stress and suppression of effector T cell activation, leading to decreased expression of adhesion molecules such as VCAM-1 and ICAM-1, dampening immune cell infiltration. Furthermore, Tub A reduces the activation of NF-κB signaling, resulting in decreased production of proinflammatory cytokines like TNF-α, IL-6, and IL-1. Overall, Tub A prevents inflammation in synovial and peripheral tissues, modulates immune responses, and alleviates autoimmune disease. Tub A. Tubastatin A; HDAC6. Histone deacetylase 6; FOXP3. Forkhead box P3; Treg. Regulatory T cells; ROS. Reactive oxygen species; VCAM-1. Vascular cell adhesion molecule 1; ICAM-1. Intercellular adhesion molecule 1; NF-κB. Nuclear factor κB; TNF-α. Tumor necrosis factor-α; IL-6. Interleukin-6; IL-1β. Interleukin-1β; HATs. Histone acetyltransferases.

Hashimoto’s thyroiditis is a chronic autoimmune condition that affects the thyroid gland, leading to hypothyroidism [154]. Chang *et al*. [155] conducted a study to evaluate the impact of Tub A (30 mg/kg via the i.p. route for 5 d) or ACY-1215 (HDAC 6 inhibitor) in an experimental autoimmune thyroiditis model, as well as in HEK293T cells, where Tub A treatment was administered at a concentration of 10 μmol/L. The results indicated that the treatment with Tub A significantly reduced the HDAC6 expression in HEK293T cells, ultimately decreasing Th17 cell differentiation through the pyruvate kinase M2 (PKM2)/STAT3 signaling pathway [155]. *In vivo*, the Tub A treatment led to a decrease in several key biochemical parameters, such as anti-thyroid peroxidase, anti-thyroglobulin, IL-17A, and interferon gamma by downregulating HDAC6 overexpression, reducing STAT3 phosphorylation, and inhibiting PKM2 nuclear translocation. Moreover, the study observed a decline in Th17 cell differentiation and an increase in TGF-β levels, which promoted Treg cell differentiation. These findings shed light on the potential future direction, which may involve further exploration of mechanisms involving HDAC6 to assess its potential as a therapeutic target for Hashimoto’s thyroiditis [155]. Therefore, Tub A shows promise in halting disease progression, especially in autoimmune conditions. However, additional research is needed to fully comprehend its molecular effects and clinical potential **(**Fig. 5**)**.

### 3.7 Pulmonary diseases

HADC6 has emerged as an important regulator in modulating inflammatory and fibrosis signaling in pulmonary diseases. Idiopathic pulmonary fibrosis is a fatal and progressive interstitial disease that involves HDAC6-dependent EMT and lysine acetylation of key regulatory proteins [156], [157]. A study demonstrated that HDAC6 inhibition by Tub A in bleomycin-induced lung fibrosis downregulated the TGF-β/PI3K/Akt pathway and TGF-β1-induced type I collagen production in pulmonary fibroblasts [158]. The results of this study are further supported by the findings of a report that suggested Tub A suppresses the proliferation of fibroblasts by inhibiting the HDAC6-dependent PI3K/Akt/mTOR pathway [159]. Acute pulmonary embolism (APE) is a pulmonary circulation condition that presents as a clinical illness due to blockage of the pulmonary artery. Zhou *et al*. [160] showed that the lungs of APE mice had significantly higher levels of HDAC6 expression. The treatment with Tub A reduced lung tissue HDAC6 expression. HDAC6 inhibition also decreased the inflammatory response induced by APE. Specifically, HDAC6 inhibition reversed the increased production of proinflammatory cytokines in APE rats, such as TNF-α, IL-1β, IL-6, and IL-18 [160]. Additionally, the lungs of APE rats showed activation of the NLRP3 inflammasome, which was inhibited by HDAC6 blockade. Furthermore, HDAC6 inhibition blocked the activation of the ERK/Akt signaling pathway, a crucial pathway involved in inflammation [160]. The findings suggest that by inhibiting HDAC6, Tub A may reduce lung dysfunction and pathological damage caused by APE by suppressing the Akt/ERK signaling pathway. Another study investigated the protective effects of Tub A on acute lung injury caused by cecal ligation and puncture. The findings demonstrated reduced lung damage and improved histological changes. Tub A also increased innate immune cells and macrophages, restoring B lymphocyte levels [161].

### 3.8 Viral diseases

ROS are generated when the human immunodeficiency virus type 1 (HIV-1) transactivator of transcription (Tat) activates NADPH oxidase, leading to neuroinflammation in the central nervous system [162]. Youn *et al*. [163] investigated the relationship between NADPH oxidase and HDAC6 in HIV-1 Tat-stimulated astrocytes and found that inhibiting HDAC6 reduced ROS production and NADPH oxidase activation. *HDAC6* knockdown also suppressed the expression of NADPH oxidase subunits, including NOX-2, p47phox, and p22phox. The selective HDAC6 inhibitor, Tub A, blocked HIV-1 Tat-induced ROS generation and NADPH oxidase activation. Additionally, *NOX-2* deletion reduced HIV-1 Tat-induced HDAC6 expression and subsequent chemokine production. These findings suggest that targeting HDAC6 and NADPH oxidase could be a potential therapeutic approach to mitigate HIV-1 Tat-induced neuroinflammation [163]. In addition, Tub A has also shown potential in suppressing the proliferation of the hepatitis C virus in human hepatocytes. The findings of a study by Kozlov *et al*. [164] demonstrated that Tub A inhibits HDAC6 activity within the dose range of 3 to 10 μmol/L, resulting in hyperacetylation of α-tubulin. This process regulates the chaperone activities of Hsp90 and Prdx1/2, which, in turn, modulate the cellular redox potential. A decrease in viral RNA concentration in infected Huh7-luc/neo hepatocyte cells following Tub A treatment indicates it as a potential therapeutic target for hepatitis C [164]. However, further mechanistic studies are required to clarify the relationship between HDAC6-mediated microtubule acetylation and hepatitis C virus replication.

### 3.9 Cushing’s disease, optic nerve diseases, and Chagas disease

Cushing’s disease, an endocrine disorder caused by hypercortisolism, is primarily driven by pituitary adenomas that produce excessive adrenocorticotropic hormone (ACTH) [165]. This increased ACTH secretion stimulates the adrenal glands to produce excess cortisol, while corticotroph adenoma cells disrupt the normal cortisol feedback mechanism. Pan-HDAC inhibitors have been found to reduce cell proliferation and ACTH production in AtT-20 corticotroph tumor cells [166]. According to Hagiwara *et al*. [167], Tub A selectively decreased the expression of pro-opiomelanocortin (*Pomc*) and pituitary tumor transforming gene 1 (*Pttg1*) mRNA in AtT-20 murine corticotroph tumor cells while increasing levels of phosphorylated Akt, ultimately preventing tumor development.

The progressive loss of photoreceptors is a hallmark of retinal disorders such as age-related macular degeneration and retinitis pigmentosa. HDAC6 has been identified as a potential target for neuroprotection and regeneration [168]. Leyk *et al*. [168] demonstrated that HDAC6 is constitutively present in the 661 W cone-like mouse cell line and the mouse retina. Inhibition of HDAC6 with Tub A led to acetylation of α-tubulin, activation of heat-shock transcription factor 1, and improved cell survival after oxidative stress. Additionally, HDAC6 suppression altered the redox regulatory protein Prdx1 in response to oxidative stress. In the zebrafish model of hereditary sight loss, the treatment with Tub A restored retinal morphology and visual function, highlighting the potential of HDAC6 inhibition in protecting retinal cells from oxidative damage [168].

*T. cruzi*, the causative agent of Chagas disease, is a significant public health concern globally [169]. The parasite undergoes aberrant epigenetic processes, including post-translational modifications like phosphorylation, methylation, and acetylation. These modifications are crucial in various cellular functions, such as gene expression, transcription, and replication [170]. Santos *et al*. [171] have shown that Tub A selectively inhibits HDAC6 expression, leading to increased levels of acetylated tubulin in *T. cruzi* that affected the parasite growth and its microtubule dynamics. These findings suggest that HDAC6-dependent regulation of the cytoskeleton may represent a potential therapeutic target in Chagas disease.

## 4 Current perspectives and future directions

**Fig. 6:**
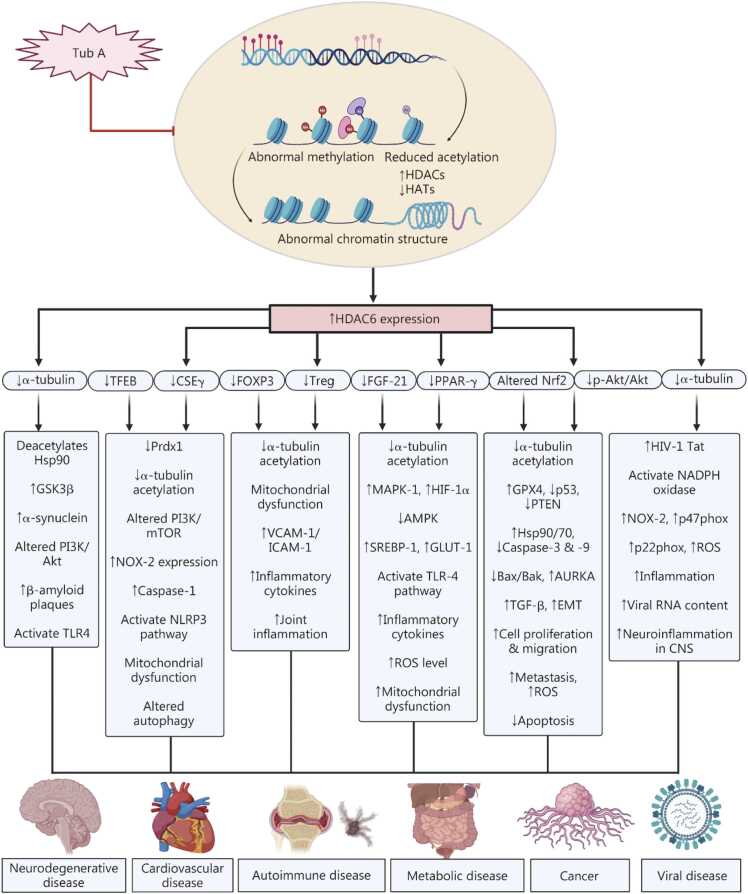
Effect of Tub A on multiple diseases. By selectively inhibiting HDAC6, Tub A increases the acetylation of α-tubulin and helps regulate the activity of Hsp90. This process results in lower levels of GSK3β and α-synuclein, influencing the PI3K/Akt signaling pathway, reducing β-amyloid plaques. Consequently, Tub A decreases TLR4 activation and lowers cytokine levels, targeting neurodegenerative diseases. Further, Tub A enhances anti-oxidant defences through the upregulation of Prdx1, the suppression of NOX-2, and the improvement of autophagy. It also modulates the PI3K/mTOR pathway and reduces NLRP3 activation, mitigating cardiovascular diseases. In addition to enhancing α-tubulin levels, Tub A restores FOXP3 regulatory T cell levels. It ultimately reduces proinflammatory cytokine release and alleviates joint inflammation, serving as a potential target for autoimmune diseases. Moreover, Tub A upregulates FGF-21/AMPK signaling, restores GLUT-1 function, reduces ROS, and decreases proinflammatory cytokine levels, preventing mitochondrial dysfunction and attenuating different types of metabolic diseases. Further, Tub A enhances the α-tubulin acetylation, and tumor suppressor activity of p53, and PTEN while modulating key pathways of PI3K/Akt and hedgehog to induce apoptosis, ferroptosis, and autophagy, and suppress proliferation, metastasis, and tumor progression. The multi-targeted mechanisms of Tub A show therapeutic potential for treating various types of cancer via downregulation of HDAC6. Additionally, Tub A enhances α-tubulin acetylation, decreases HIV-1 Tat-induced neuroinflammation, lowers viral RNA levels, and suppresses NADPH oxidase activity, ultimately reducing neuroinflammation. This pathway shows that Tub A has therapeutic potential to mitigate viral diseases. Tub A. Tubastatin A; HDAC6. Histone deacetylase 6; Hsp90. Heat shock protein 90; GSK3β. Glycogen synthase kinase 3β; TFEB. Transcription factor EB; CSEγ. Cystathionine γ-lyase; Prdx1. Peroxiredoxin 1; FOXP3. Forkhead box P3; Treg. Regulatory T cells; PPAR-γ. Peroxisome proliferator-activated receptor gamma; Nrf2. Nuclear factor erythroid 2-related factor 2; GPX4. Glutathione peroxidase 4; PTEN. Phosphatase and tensin homolog; ROS. Reactive oxygen species; MAPK. Mitogen-activated protein kinase; NF-κB. Nuclear factor κB; TLR4. Toll-like receptor 4; SREBP-1. Sterol regulatory element-binding protein 1; GLUT-1. Glucose transporter type 1; CNS. Central nervous system; HATs. Histone acetyltransferases; NADPH. Nicotinamide adenine dinucleotide phosphate hydrogen; NOX-2. NADPH oxidase 2; TGF-β. Transforming growth factor-β; AURKA. Aurora kinase A; EMT. Epithelial-mesenchymal transition; AMPK. 5’ adenosine monophosphate-activated protein kinase; HIF-1α. Hypoxia-inducible factor-1α; FGF-21. Fibroblast growth factor 21; VCAM-1. Vascular cell adhesion molecule 1; ICAM-1. Intracellular adhesion molecule 1; mTOR. Mammalian target of rapamycin.

Over the past decade, extensive research has demonstrated that Tub A is a potent and selective HDAC6 inhibitor, making it a preclinically validated therapeutic candidate for a wide range of pathological conditions [19], [25]. These include various neurological disorders, CVDs, cancer, metabolic diseases, Hashimoto’s thyroiditis, and others **(**Fig. 6**)**. However, it is worth noting the bidirectional regulation of the apoptotic mechanism by Tub A in cancer and PAH by acting on cytosolic targets in a condition-specific manner. For instance, in cancer cells, by inhibiting HDAC6, Tub A increases Ku70 acetylation, disrupting its binding to pro-apoptotic Bax, thus translocating it into mitochondria [76], [172]. This process results in activation of caspase-dependent apoptosis. Simultaneously, Tub A also acetylates Hsp90 and α-tubulin, destabilizing oncogenes, leading to further increased apoptosis [76], [117]. Moreover, α-tubulin acetylation by Tub A leads to microtubule stabilization, preserving the endothelial barrier integrity, and favouring cell survivability over apoptosis. Consequently, the same upstream HDAC6 inhibition can be pro-apoptotic in cancer cells but cytoprotective in PAH endothelium [76].

Although Tub A is still in pre-clinical testing, the United States Food and Drug Administration (FDA) has approved several HDAC inhibitors, including Vorinostat, Panobinostat, and Romidepsin, for clinical use, primarily in hematologic malignancies [173]. These compounds, varying in structure, such as hydroxamic acids and benzamides, exhibit zinc-binding activity essential for HDAC inhibition. Notably, selective HDAC6 inhibitors such as ACY-1215 and ACY-241 have advanced to clinical trials, offering potential avenues for developing more targeted and effective therapies [16], [174]. Compared with other HDAC6 inhibitors, Tub A shows very high selectivity toward HDAC6. It mainly affects cytoplasmic substrates such as α-tubulin rather than global histone acetylation. However, Tub A has a high efflux ratio, short half-life, low oral exposure, and poor BBB penetration [22]. In contrast, ACY-1215, ACY-241, and KA2507 show improved oral bioavailability and longer systemic exposure. Moreover, CKD-L, which has been investigated for arthritis, has better BBB penetration than Tub A [33], [34], [35], [36], [151]. Despite these pharmacokinetic limitations, Tub A remains widely used because of its strong isoform selectivity and lower off-target effect, making it a useful reference compound for studying HDAC6-dependent mechanisms. Recent approaches, such as nanoformulation or nanoloading, would help to address its efflux-related limitations.

HDAC6 inhibition by Tub A can mitigate the progression of several chronic diseases by attenuating fibrosis, oxidative stress, and inflammation through the downregulation of key proinflammatory and profibrotic mediators, including TGF-β, NLRP3, IL-1β, TNF-α, and phosphorylated p38 MAPK [153]. Furthermore, Tub A has been reported to alleviate dyslipidemia, obesity and diabetic conditions, and regulate adipokine levels, through the upregulation of FGF-21 [25], [27], [29], [153], [175]. Further, targeting the HDAC6/HDAC8/PKM2 signaling pathway through Tub A could represent a new therapeutic approach for several diseases. To date, no clinical studies evaluating the safety and efficacy of Tub A in human subjects have been conducted. Future studies should also focus on more detailed mechanisms identifying how Tub A preferentially modulates distinct downstream pathways, such as microtubule dynamics, inflammasome signaling, mitochondrial function, or autophagy, in different disease settings. A systematic evaluation of tissue-specific target engagement and pathway cross-talk will be essential to better integrate these disease-specific mechanisms and strengthen the translational relevance of Tub A. Although Tub A has been evaluated across different pathological settings, it has been most extensively studied in neurodegenerative and cancer models, with limited preliminary findings in other conditions. This underscores the importance of further research into Tub A HDAC6’s inhibition in disease processes.

Moreover, a report also suggests that Tub A, when administered alone at all tested concentrations, did not induce any toxicity [30]. To facilitate the clinical translation of Tub A, future studies should integrate advanced nano-delivery strategies with detailed mechanistic and pharmacokinetic evaluations while addressing key challenges such as human safety data, species-specific variations in HDAC6 biology, potential off-target effects, and long-term toxicity, which may ultimately improve therapeutic applicability and translational potential.

## 5 Conclusions

HDAC6, the largest member of the HDAC family, is primarily found in the cytoplasm and plays a crucial role in regulating cellular dynamics, maintaining proteostasis, and supporting survival pathways. However, dysregulated or overexpressed HDAC6 activity has been linked to increased inflammatory responses and oxidative stress, which contribute to the progression of various diseases. Tub A, a highly selective HDAC6 inhibitor, has attracted considerable interest in preclinical studies for treating conditions associated with elevated HDAC6 levels, including neurodegenerative diseases, metabolic disorders, cancers, and more. Despite promising results at the preclinical level, Tub A has not yet progressed to clinical trials due to limitations in pharmacokinetics and pharmacodynamics. Its high efflux and low permeability across the BBB require higher doses of Tub A, which may result in potential off-target effects. Recent studies involving nanoformulation and nanoloading of Tub A have indicated that optimized formulations can address these pharmacokinetic and pharmacodynamic challenges, supporting its future development for clinical applications.

## Abbreviations

AβAmyloid β

ACTHAdrenocorticotropic hormone

ADAlzheimer’s disease

AP-1Activator protein-1

APEAcute pulmonary embolism

AUCArea under curve

BBBBlood-brain barrier

ChATCholine acetyltransferase

CMAChaperone-mediated autophagy

CSEγCystathionine γ-lyase

CVDsCardiovascular diseases

DMDDuchenne muscular dystrophy

EGFREpidermal growth factor receptor

ERKExtracellular signal-regulated kinase

HDACHistone deacetylase

H/RHypoxia/reoxygenation

HFHeart failure

HIV-1Human immunodeficiency virus type 1

Hsp90Heat shock protein 90

FGF-21Fibroblast growth factor 21

GBMGlioblastoma multiforme

GPX4Glutathione peroxidase 4

GSK3βGlycogen synthase kinase 3β

ILInterleukin

IRIonizing radiation

LPSLipopolysaccharide

MAPKMitogen-activated protein kinase

MI/RMyocardial ischemia/reperfusion

mTORMammalian target of rapamycin

NADPHNicotinamide adenine dinucleotide phosphate hydrogen

NF-κBNuclear factor κB

NLRPNucleotide-binding oligomerization domain, leucine-rich repeat, and pyrin domain containing 3

NOX-2NADPH oxidase 2

6-OHDA6-hydroxydopamine

PAH Pulmonary arterial hypertension

PD Parkinson’s disease

PdNPsPalladium nanoparticles

PGCLPoly(glycolide-co-ε-caprolactone)

PKM2Pyruvate kinase M2

PrdxPeroxiredoxins

ROSReactive oxygen species

SCISpinal cord injury

SFSilk fibroin

Sig1RSigma-1 receptor

STAT3Signal transducer and activator of transcription 3

TatTransactivator of transcription

TGF-βTransforming growth factor-β

TKNPThioketal nanoparticles

TLR4Toll-like receptor 4

TNF-αTumor necrosis factor-α

Tub ATubastatin A

XBP1X-box binding protein 1

## Ethics approval and consent to participate

Not applicable.

## Authors’ contributions

UN, RW, and AK conceived the study, coordinated it, and helped draft the manuscript. SR and SKS drafted the manuscript, designed the figures, and substantively revised both. PY and AK contributed to editing the manuscript. SR, SKS, and AK helped revise the manuscript. All the authors read and approved the final manuscript.

## Funding

This work was supported by the Indo-German Science and Technology Centre (IGSTC/PECFAR/Call 2022/USAK/36/2022-23/Award letter/488) for providing the Paired Early Career Fellowship in Applied Research (PECFAR) fellowship. We also extend our thanks to Bayer Pharmaceuticals (application ID: PHD-2023-10682) for providing the Medha fellowship. We also express our gratitude to the Indian Council of Medical Research (ICMR) (project ID-2021-8630), Government of India, for providing an SRF fellowship. The authors would also like to appreciate the support from Anusandhan National Research Foundation (ANRF) under the Partnerships for Accelerated Innovation and Research (PAIR) programme under the sanction order No. ANRF/PAIR/2025/000029/PAIR dated 20 September 2025 to Central University of Punjab.

## Competing interests

The authors declare that they have no competing interests.

## Data Availability

Not applicable.
